# Gastric band is safe and effective at three years in a national study subgroup of non-morbidly obese patients

**DOI:** 10.3325/cmj.2014.55.405

**Published:** 2014-08

**Authors:** Goran Ribaric, Jane Buchwald

**Affiliations:** 1 European Surgical Institute, Ethicon Endo-Surgery (Europe), Hamburg, Germany; 2 Division of Scientific Writing, Medwrite Medical Communications, Maiden Rock, WI, USA

## Abstract

**Aim:**

To analyze the 3-year outcomes of lower body mass index (BMI) (<35 kg/m^2^) adjustable gastric band (AGB) recipients across multiple sites in the French health insurance system.

**Methods:**

From prospectively collected data on a cohort of 517 morbidly obese Swedish Adjustable Gastric Band^®^ (SAGB) patients (Clinical Trials Web database, #NCT01183975), a retrospective analysis of a subgroup of 29 low-BMI patients was conducted. Patients had a severe obesity-related comorbidity, had undergone a prior bariatric procedure requiring reintervention, or had a maximum adult BMI≥40. Safety (mortality, adverse events) and effectiveness (BMI change, excess weight loss [EWL, %], total body weight loss [%TBWL], quality of life [QoL], and comorbidities) were evaluated.

**Results:**

Multiple surgical teams/sites enrolled patients and performed SAGB procedures between September 2, 2007 and April 30, 2008. Of 29 low-BMI patients (mean age, 41.3 ± 10.3 years), 89.7% were female, and obesity duration was 13.6 ± 7.3 years. Mean BMI was 31.5 ± 3.7; there were 37 comorbidities in 15/29 patients. At 3-year follow-up, BMI was 29.4 ± 4.9 (mean change, -2.3 ± 6.2; *P* = 0.069); total cohort EWL, 7.3 ± 74.8%; TBWL, 6.2 ± 18.8%; BMI≥30 to <35 EWL, 38.8 ± 48.0%; there were 7 comorbidities in 15/29 patients (*P* < 0.031). There were 20 adverse events in 13 patients (44.8%); SAGBs were retained in 25/29 (86.2%) at 3 years.

**Conclusions:**

In a retrospective analysis of a subgroup of BMI<35 kg/m^2^ patients, some following a prior bariatric procedure, SAGB was found to be safe and effective at 3-year follow-up.

For over 2 decades, since publication of the 1991 National Institutes of Health (NIH) consensus conference statement (1), the cutoff point for bariatric surgery has been morbid obesity (body mass index [BMI, kg/m^2^]≥40 or ≥35 with comorbidities), also termed class II obesity by the World Health Organization (WHO) (2). This demarcation of access to bariatric surgery was based on the observation that an increase in BMI leads to an increase in the risk of comorbid illness and premature death. Yet, investigation of the potential value of bariatric surgery as a safe and effective treatment for overweight (BMI 25-<30) and obesity class I (BMI≥30 to <35) patients has been under way since the publication of the NIH statement. In 1992 and 1995 landmark studies (3,4), Pories et al theorized that bariatric procedures might be safe and as beneficial for weight loss and comorbidity reduction in non-morbidly obese patients as it was in the morbidly obese (5). In the last half decade, the least-invasive, lowest-risk restrictive procedures, such as adjustable gastric banding (AGB), have been employed at the forefront of exploring surgical options for the <35 BMI patient.

Adjustable gastric banding comprised the vast majority, nearly 90%, of bariatric procedures performed in morbidly obese patients in France prior to 2008 (6). To assess the national social insurance-supported use of the Swedish Adjustable Gastric Band (SAGB) (7,8), the French government commissioned a prospective, 31-center, “real-life,” observation of SAGB safety and effectiveness in class II and III obese patients (9). Between September 2, 2007 and April 30, 2008, patients were selected and underwent SAGB implantation in rural and urban centers. SAGB weight-loss effectiveness analyzed on an intent-to-treat basis at the 3-year study endpoint was comparable to that of AGB findings summarized by global meta-analyses (10,11). Under the “real-world” SAGB study protocol requirement of consecutive recruitment and surgeon discretion, 29 patients (5.6% of 517) were included in the national SAGB study who presented with a BMI<35 and a severe obesity-related comorbidity, and/or had experienced a prior complicated bariatric surgery requiring revision, and/or had previously sustained a maximum adult BMI≥40. With the aim of contributing safety and effectiveness findings to the growing <35 BMI evidence base, we report 3-year outcomes for the French low-BMI SAGB study group.

## Methods

### Study protocol

During 2007, the French Health Technology Assessment Body (HAS) (12,13) requested that Ethicon Endo-Surgery (Europe) GmbH sponsored and performed a countrywide health insurance study to assess reimbursement of the SAGB product in France. The study was registered (Clinical Trials Web database, #NCT01183975) (14) and a sponsor-developed protocol and case report form developed to direct implementation of HAS requirements and good clinical practices (GCPs) (ie, patient welfare in study design, ethical study conduct), defined by ISO EN 14155-1 and -2 (15,16). Ethical approval and protocol approval were given by HAS, the Commission Nationale de l'Informatique et des Libertés, and the Comité Consultatif sur le Traitement de l’Information en matičre de Recherche dans le domaine de la Santé. Treatment payments were covered by national health insurance (13).

A contract research organization, Medextens SARL, Paris, France, and an independent monitoring committee consisting of a non-participating bariatric surgeon, a pharmacologist, and a medical nutritionist supervised the study’s progress and prepared an interim report for review by HAS and the sponsor. Patients were required to provide written informed consent before surgery per Declaration of Helsinki (17) and GCP guidelines.

### Design and setting

The prospective, multicenter, noncomparative study design aimed to facilitate observation and reporting of outcomes in a morbidly obese study cohort, of which the current low-BMI cohort was a subgroup (9). Primary HAS objectives were to asses SAGB safety (mortality, adverse event [AE] occurrence) and clinical effectiveness (changes in weight loss, quality of life [QoL], comorbid illness) in varied French hospital settings.

In order to incorporate “real-life” practice experiences across geographically diverse regions of France, surgeons were selected from academic, private, and public institutions with differing bariatric surgery volumes. Per GCP standards, surgeons were required to undergo training in the protocol; selected surgeons recruited SAGB patients consecutively.

### Inclusion criteria

Study eligibility for the primary trial was based on a recruitment goal of >500 patients with <20% loss to follow-up after 3 years. General inclusion criteria stipulated patients with morbid obesity after failed medical treatment and no contraindications in accord with French (12), European (18), and American NIH bariatric surgery guidelines (1). French residents with a BMI<35 were permitted inclusion in the consecutive SAGB study enrollment if they had an adult maximum BMI≥40, and/or had a severe obesity-related comorbidity (thus, were receiving SAGB as a primary intervention in the current study [an “index SAGB”]), or if they required reintervention following a complicated prior bariatric procedure [a “PBP+SAGB”] (SAGB as a secondary intervention).

### Variables

Safety variables analyzed for the low-BMI cohort were mortality and frequency of AEs. Effectiveness variables were evaluated as change over 3 years in absolute weight (AW); BMI; excess body weight (EW); percentage EW loss (%EWL), ie, baseline AW – follow-up AW/EW, calculated by Miller’s formulas (19-23) for identifying ideal weight, corresponding to the midpoint value of the medium-frame range on the Metropolitan Life Insurance Height and Weight Tables ×100; and percentage total body weight loss (%TBWL), (ie, baseline AW – follow-up AW/baseline AW × 100) (24). Health-related QoL and changes in comorbid illness were also analyzed.

### Quality of life instruments

The generic EuroQoL 5-Dimensions (EQ-5D), a psychometric instrument valued for its utility in calculating quality adjusted life years as well as the relative cost-effectiveness of obesity interventions, such as AGB (25), was used as a measure of QoL. The EQ-5D is a health-related QoL evaluation with 5 items and a visual analogue scale (EQ-VAS) (26-29) that provides a 5-dimensional profile: mobility, usual activities, self-care, anxiety/depression, and pain/discomfort. Dimensions are presented as 1 item with 3 response options: severe problems, some problems, and no problems. Item responses can be weighted normatively to derive a utility score (range -0.594 to 1, where 1 = ultimate health). A clinically important difference has been identified at ≥0.07 on the EQ-5D scale (29). The EQ-VAS module is a single-item global QoL evaluation in which patients rate their current health (scale from 0 = worst imaginable to 100 = best imaginable) (30).

### Data collection

Protocol-prescribed safety and weight data collection and assessment measures were the only standardized requirements for the surgical centers, per the “real-life” observational study design. Baseline characteristics (eg, gender, age) were collected; weight, obesity-related comorbid disease, and QoL were recorded on the day of surgery, and at 1, 3, 6, 12, 18, 24, and 36 months postoperatively. Comorbidity data were sought via questionnaire; diagnoses were established and recorded consonant with individual investigators’ typical practice via the password-protected Medextens-Medalliance eCRF Manager (v.1.3) web database.

For the current low-BMI subgroup study, data were retrieved from the original HAS archive and sorted by script for the known 29 target patients. Coded variables that addressed identified study topics were chosen and manually exported to a dedicated SPSS database.

### Technique

SAGB procedures were performed via *pars flaccida* technique (31), and band adjustments were accomplished at the discretion of the surgeons. Three SAGB model options were available: 2200-X Quick-Close; 2100-X (with locking ring and injection port); and the BD2XV Quick-Close with Velocity^TM^ injection port.

### Statistical analysis

Statistical analyses were performed using the SPSS^®^ software package (ver. 20, IBM SPSS, Chicago, IL, USA). Quantitative demographic variables were generally reported as median and interquartile range (IQR); qualitative variables (demographic and outcome) were reported as number and percentage. Adverse events were also reported as number and percentage. Quantitative measures of change from baseline at 3 years were analyzed using the related-samples Wilcoxon signed rank test; between-group comparisons were made with the Mann-Whitney U test. The Fisher exact test was used to investigate relationships between qualitative variables. Multivariate modeling, linear regression, and logistic regression were used to explore relationships between patient characteristics, weight loss, and QoL. Alpha was set at *P* < 0.05.

## Results

Screening and enrollment of patients occurred between September 2, 2007 and April 30, 2008. The last follow-up visit at 3 years, due on April 30, 2011, was extended to November 20, 2011 to accommodate patients’ schedules. All low-BMI cases were treated laparoscopically using *pars flaccida* technique and port fixation with no conversions to laparotomy.

### Baseline patient characteristics

The SAGB BMI<35 sample consisted of 89.7% (N = 26) female and 10.3% (N = 3) male patients with a median age of 38.8 years, obesity duration of 12.0 years, AW of 87.0, EW of 28.5, and median BMI of 33.1 (Table 1). Nine (31%) patients had a baseline BMI<30 and 20 (69%) had a BMI≥30 to <35.

**Table 1:** Preoperative patient characteristics*

Characteristic	Median (IQR), N = 29
Gender:	
Male, N (%)	3 (10.3)
Female, N (%)	26 (89.7)
Age (yrs)	38.8 (33.9*-*50.4)
Duration of obesity (yrs)	12.0 (10.0*-*17.5)
Height (m)	1.7 (1.6*-*1.7)
AW (kg)	87.0 (76.0*-*94.5)
Ideal body weight (kg)^†^	60.9 (58.2*-*63.3)
EW (kg)	28.5 (16.3*-*33.0)
BMI (kg/m^2^):	33.1 (28.8*-*34.6)
<30, N (%)	9 (31.0)
≥30 and <35, N (%)	20 (69.0)
Intervention:	
PBP + SAGB^‡^, N (%)	17 (58.6)
Index SAGB, N (%)	12 (41.4)
At least 1 comorbidity, N (%)	15 (51.7)
History of family obesity, N (%)	19 (65.5)
EQ-5D	0.7 (0.3*-*0.8)
EQ-VAS	50.0 (40.0*-*74.0)

*Abbreviations: IQR *–* interquartile range; BMI – body mass index; AW – absolute weight; EW – excess weight; PBP – Prior bariatric procedure before Swedish Adjustable Gastric Band [SAGB] implantation in current study; index SAGB – SAGB as first and only bariatric procedure; EQ-5D – EuroQoL 5-Dimensions; EQ-VAS – EuroQoL-Visual Analogue Scale.

†Ideal body weight derived from the Metropolitan Weight Tables for Life Insurance, 1983.

‡SAGB implant in this study was either an index intervention, or a SAGB following a complicated prior bariatric procedure (PBP) before the current study. 58.6% of the BMI<35 cohort were reintervention patients that satisfied the consecutive recruitment condition.

Fifty-two percent (15/29) of patients presented with at least one comorbidity. Median EQ-5D was 0.7 and median VAS, 50.0. A history of family obesity was reported in 19 patients (65.5%). SAGB was the first bariatric surgery in 12 patients (41.4%), referred to subsequently as index SAGB patients, and a reintervention following a prior bariatric procedure that involved serious complications in 17 (58.6% PGP+SAGB patients). Fifteen of 17 PBP+SAGB patients indicated that they had undergone prior AGB, while 1 reported prior sleeve gastrectomy, and 1, prior gastric balloon; 15/17 reported having had good results (ie, weight loss and comorbidity reduction) before experiencing poor weight loss and a variety of complications and subsequently selecting SAGB as their reintervention treatment. This subjective reporting was corroborated by baseline data analysis that indicated that PBP+SAGB patients, compared to index SAGB patients, had a significantly lower median (IQR) number of comorbidities (0.0 [0.0-0.5] vs 2.0 [1.3-4.0]; *P* = 0.001), significantly higher global QoL [EQ-VAS] (70.0 [50.0-80.0] vs 40.0 [25.0-55.0]; *P* = 0.003), and significantly lower median BMI (30.1 [27.7-33.2] vs 34.6 [34.0-34.8]; *P* = 0.002). Indeed, 8/9 BMI<30 SAGB patients (89%) were PBP+SAGB patients; whereas 45% (9/20) of BMI≥30 to <35 SAGB patients were PBP+SAGB patients. With respect to results and interpretation presented herein, BMI<35 PBP+SAGB patients were, largely, former class-III (≥40 kg/m^2^) morbidly obese patients. Maximum adult BMI for PBP+SAGB patients was significantly greater than that for index SAGB patients (40.4 [38.1-42.6] vs 35.8 [34.2-37.5]; *P* = 0.001).

### Adverse events

Fifty-five percent (16/29) of SAGB patients presenting with a BMI<35 experienced no adverse events (AEs) over 3-year follow-up. There was an 86.2% overall rate of band survival, that is, bands that remained implanted. There were 20 confirmed AEs in 13 patients (44.8%): 1 in 7 patients (24.1%); 2 in 5 patients (17.2%); and 3 in 1 patient (3.5%). An overall rate of 0.23 confirmed adverse events per patient-year was observed. Confirmed AEs in order of frequency were: band removal 4 (14%), port rotation 3 (10.3%), band slippage 2 (7%), esophageal dilation 2 (7%), food intolerance 2 (7%), abdominoplasty 2 (7%), dysphagia 1 (3.5%), GERD 1 (3.5%), port malposition 1 (3.5%), port reintervention (no removal) 1 (3.5%), and port dysfunction/removal 1 (3.5%). PBP+SAGB patients had a significantly higher median number of AEs than index SAGB patients (1.0 [0.0-2.0] vs 0.0 [0.0-0.0]; *P* = 0.030). In fact, 17/20 (85%) confirmed AEs, and all 4 confirmed band removals (ablations), occurred in the PBP+SAGB group. Conversely, 83.3% (10/12) of index-SAGB patients experienced no AE.

### Weight loss

**Figure 1:**
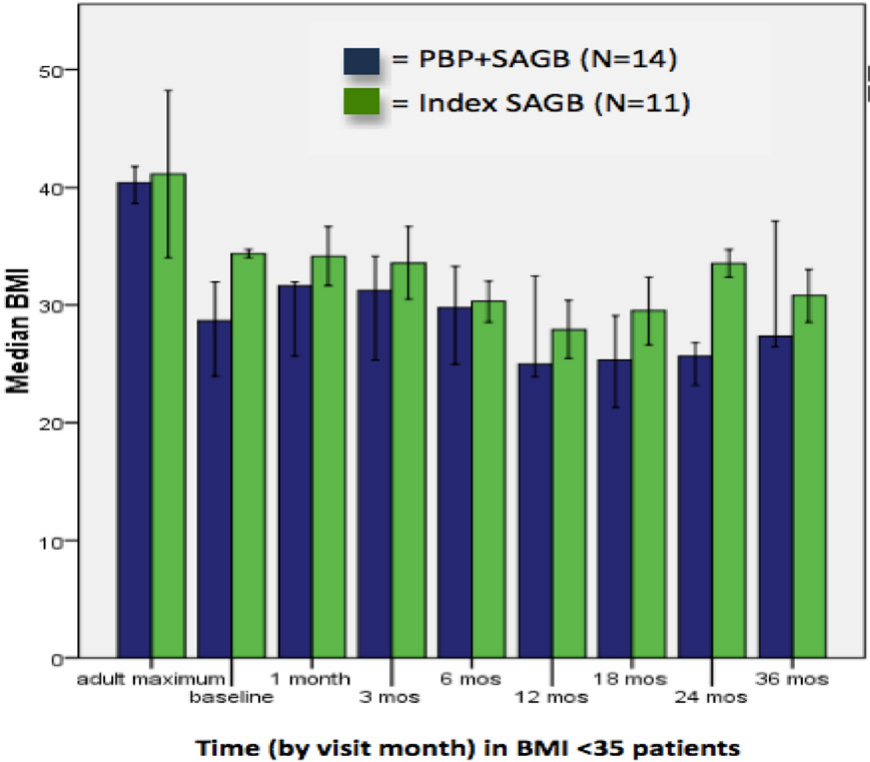
Evolution in median body mass index (BMI, kg/m^2^) over 3 years in Swedish Adjustable Gastric Band (SAGB) cohort with baseline BMI<35 as moderated by intervention type (prior bariatric procedure [PBP+SAGB] vs first intervention [index SAGB]). Error bars represent the ~ 95% confidence interval bracketing the median.

Three-year postoperative weight outcomes for BMI<35 SAGB patients were available in 86.2% (25/29) of patients. Median AW was 80.0 (72.0-88.0) compared to 87.0 (76.0-94.5) at baseline (Table 2), representing a median AW reduction of 3.0 (-5.5-16.5: *P* = 0.126), corresponding to a median %TBWL of 2.9 (-6.6-17.5). Median EW was 22.1 (10.0-26.9) compared to 28.5 (16.3-33.0) at baseline, representing a median EW reduction of 3.0 (-5.5-16.5: *P* = 0.126), corresponding to a median %EWL of 8.8 (-28.7-54.3). Median BMI was 30.1 (25.9-32.9), compared to a preoperative median BMI of 33.1 (28.8-34.6). This change represented an overall median BMI reduction of 1.0 (-2.0-6.0; *P* = 0.123). Median BMI evolution over 3 years by type of SAGB intervention (PBP+SAGB vs index SAGB) for the BMI<35 cohort is presented in Figure 1.

**Table 2:** Weight loss*†

Total group	Median (IQR), N = 25			
	Preoperative	3-y	Median change	*P*-value^‡^
AW (kg)	87.0 (76.0-94.5)	80.0 (72.0-88.0)	3.0 (-5.5-16.5)	0.126
BMI (kg/m^2^)	33.1 (28.8-34.6)	30.1 (25.9-32.9)	1.0 (-2.0-6.0)	0.123
EW (kg)	28.5 (16.3-33.0)	22.1 (10.0-26.9)	3.0 (-5.5-16.5)	0.126
TBWL (%)	—	2.9 (-6.6-17.5)	—	—
EWL (%)	—	8.8 (-28.7-54.3)		—
**Subgroup 1 comparison**				
**PBP+SAGB (n = 14)**				
AW (kg)	82.0 (74.5-89.3)	85.0 (78.0-90.8)	-4.0 (-9.3-5.8)	0.401
BMI (kg/m^2^)	31.0 (28.0-33.1)	31.6 (29.0-33.3)	-1.6 (-3.2-2.0)	0.421
EW (kg)	22.6 (13.5-29.3)	24.6 (17.6-28.6)	-4.0 (-9.3-5.8)	0.401
TBWL (%)	—	-5.0 (-12.5-6.1)	—	—
EWL (%)	—	-20.0 (-68.8-18.6)	—	—
**Index SAGB (n = 11)**				
AW (kg)	93.0 (87.0-100.0)	76.0 (65.0-83.0)	16.0 (3.0-31.0)	0.010
BMI (kg/m^2^)	34.5 (34.0-34.8)	28.5 (23.0-32.9)	5.9 (1.0-11.0)	0.010
EW (kg)	32.1 (29.4-34.1)	15.3 (2.5-22.1)	16.0 (3.0-31.0)	0.010
TBWL (%)	—	17.0 (2.9-32.3)	—	—
EWL (%)	—	51.1 (8.8-92.4)	—	—
**Subgroup 2 comparison**				
**BMI<30 (n = 7)**				
AW (kg)	76.0 (68.0-85.0)	85.0 (80.0-88.0)	-9.0 (-15.0 to -4.0)	0.042
BMI (kg/m^2^)	26.9 (25.7-28.7)	30.1 (27.4-33.4)	-3.2 (-5.0 to -1.7)	0.042
EW (kg)	13.5 (13.1-15.9)	22.5 (15.9-33.1)	-9.0 (-15.0 to -4.0)	0.042
TBWL (%)	—	-11.8 (-17.6 to -5.9)	—	—
EWL (%)	—	-66.5 (-153.3 to -28.3)	—	—
**BMI≥30 (n = 18)**				
AW (kg)	90.0 (85.3-97.0)	78.0 (67.3-90.8)	10.5 (-0.5-19.8)	0.006
BMI (kg/m^2^)	34.0 (32.3-34.6)	29.6 (25.3-32.9)	3.7 (-0.2-7.6)	0.006
EW (kg)	31.1 (25.8-33.7)	20.0 (7.1-26.8)	10.5 (-0.5-19.8)	0.006
TBWL (%)	—	11.0 (-0.7-23.0)	—	—
EWL (%)	—	33.1 (-2.5-73.8)	—	—

*Abbreviations: IQR – interquartile range; BMI – body mass index; AW – absolute weight; EW – excess weight; PBP – Prior bariatric procedure before Swedish Adjustable Gastric Band [SAGB] implantation in current study; EWL – EW loss; TBWL – total body weight loss.

†Calculations based on patients with complete preoperative and 3-y follow-up data.

‡Related Samples Wilcoxon Signed Rank Test.

**Figure 2:**
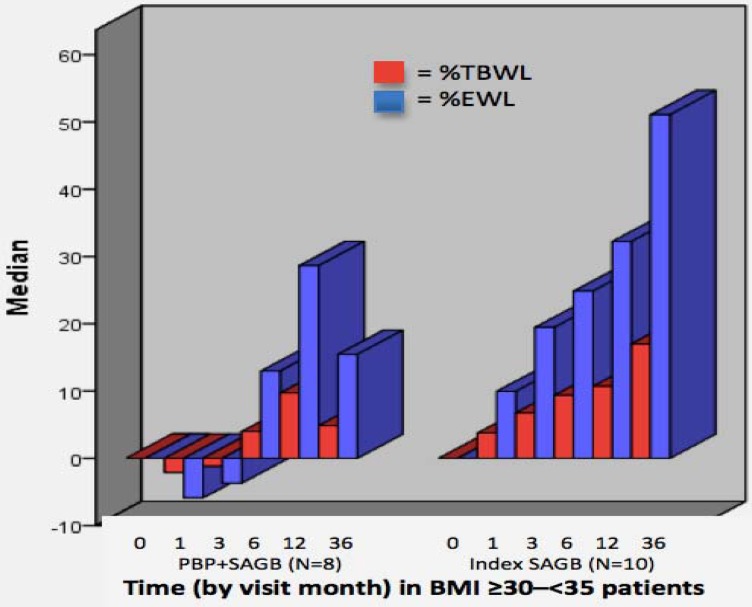
Median weight-loss trends to 3 years post Swedish Adjustable Gastric Band (SAGB) procedure for patients with preoperative body mass index (BMI, kg/m^2^)≥30 to <35 as moderated by type of operation (prior bariatric procedure [PBP+SAGB] vs first intervention [index SAGB]) expressed in percentage total body weight loss (%TBWL) and percentage excess weight loss (%EWL). Note: Follow-up rate at 18 and 24 months was not sufficient for reliable assessment.

Total cohort median changes in weight-related obesity indicators were not significant at 3 years; however, a high level of individual variation in weight-loss outcomes was noted. While some BMI<35 patients lost significant weight, some gained weight as indicated by negative %TBWL and %EWL values. Subgroup analyses, by intervention type and BMI category, were carried out. As detailed in Table 2, index SAGB patients experienced significantly greater median %EWL than PBP+SAGB patients (51.1 [8.8-92.4] vs -20.0 [-68.8-18.6]; *P* = 0.001). In fact, while PBP+SAGB patients actually gained a median 4.0 kg of AW over 3 years, corresponding to a BMI increase of 1.6 kg/m^2^, index SAGB patients experienced significant changes over time in median weight-loss indicators: AW decreased by 16 kg (*P* = 0.010), corresponding to a BMI decrease of 5.9 kg/m^2^ (*P* = 0.010). Finally, patients with a baseline BMI≥30 to <35 had significantly greater median %EWL at 3 years than did those with a BMI<30 (33.1 [-2.5-73.8] vs -66.5 [-153.3 to -28.3]; *P* = 0.001). The BMI≥30 to <35 patient subset was further subdivided into PBP+SAGB (N = 8) vs index SAGB patients (N = 10). Figure 2 depicts the evolution of %TBWL and %EWL for BMI≥30 to <35 patients as moderated by whether they were a reintervention or index SAGB. PBP+SAGB patients experienced somewhat irregular median weight outcomes over time; whereas, index SAGB patients exhibited progressive, sustained weight loss: At 3 years, PBP+SAGB median BMI was reduced by 1.3 (-1.1-4.7, *P* = 0.030), median %TBWL was 3.9 (-3.1-14.8), median %EWL was 11.8 (-8.8-49.0); index SAGB median BMI was significantly reduced by 6.1 (0.5-12.1, *P* = 0.001), median %TBWL was 17.4 (1.5-35.4), median %EWL was 51.0 (4.1-100.4).

**Figure 3:**
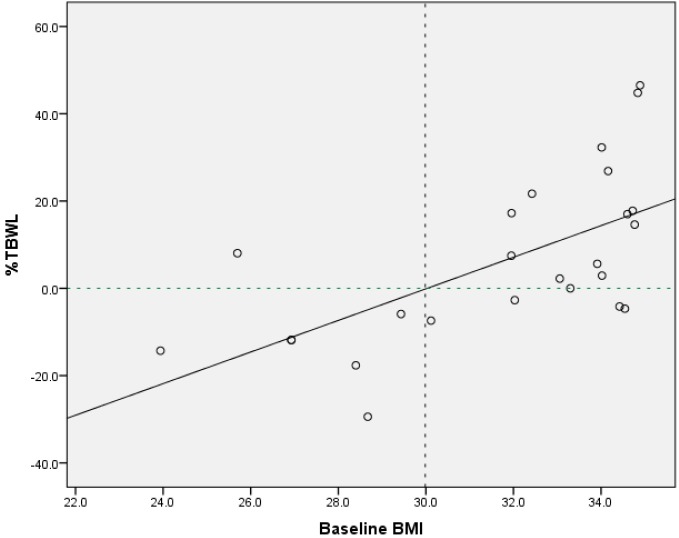
Scatter plot and regression line illustrating direct relationship between preoperative body mass index (BMI, kg/m^2^) and percentage total body weight loss (%TBWL) for BMI<35 patients following Swedish Adjustable Gastric Band (SAGB) procedure at 3 years. Intersecting reference lines represent the point on the BMI axis (BMI = 30) above which a positive %TBWL is predicted to occur at 3-year SAGB follow-up.

**Figure 4:**
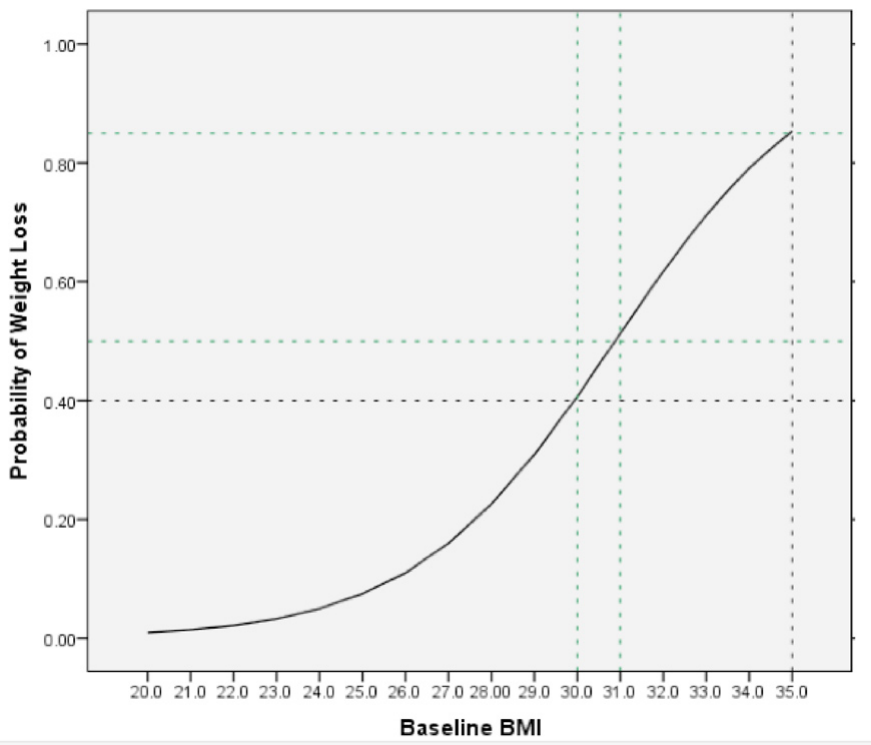
Probability curve depicting the likelihood of a patient with a given preoperative body mass index (BMI, kg/m^2^) to experience weight loss (ie, positive percentage total body weight loss, %TBWL) at 3 years after Swedish Adjustable Gastric Band (SAGB) procedure. Intersecting reference lines represent 3 sample patients with baseline BMIs of 30.0, 31.0, and 34.9 whose corresponding probability of weight loss at 3 years following SAGB procedure are calculated to be 0.40, 0.50, and 0.86, respectively.

In the development of a multivariate regression model exploring preoperative clinical variables significantly related to %TBWL (ie, presence of comorbidity [r = 0.493, *P* = 0.012], QoL [EQ-VAS] [r = -0.482, *P* = 0.027], type of SAGB operation (prior bariatric procedure or index SAGB procedure) [r = 0.591, *P* = 0.002], and BMI [r = 0.631, *P* = 0.001]), only baseline BMI was found to be an independent predictor of 3-year %TBWL in the BMI<35 SAGB cohort. Results of simple linear regression of %TBWL on baseline BMI in the form of a scatterplot and regression line are presented in Figure 3. Baseline BMI and 3-year %TBWL correlated at r = 0.631 (*P* = 0.001). A logistic regression model using BMI as the lone predictor was shown to correctly classify 83.3% of patients into their respective “weight-loss” vs “weight-gain” groups. Logistic results are presented in the form of a probability curve in Figure 4 (BMI odds ratio = 1.53 [95% CI: 1.1, 2.2]; beta coefficient = 0.428, *P* = 0.048; model constant = -13.2, *P* = 0.008).

Overall, 56.0% (14/25) of the SAGB BMI<35 cohort with complete weight data at 3 years achieved and maintained weight loss. Ninety-three percent (13/14) of those comprising the weight-loss group were patients who presented with a BMI≥30 to <35, and 57.0% (8/14) were index SAGB patients with a baseline BMI≥30 to <35. This subset of patients was the most successful in terms of weight loss: median baseline BMI was 34.7 (34.1-34.8) at 3-year follow-up, median BMI fell significantly (7.7) to 26.7 (20.2-29.4; *P* = 0.001); %TBWL was 22.3 (15.2-41.7); %EWL was 64.5 (44.4-116.3).

### Comorbidities

Adhering to the study’s observational design, no diagnostic tests for comorbidity assessment were required. At each visit, comorbidities were reported as present or absent. Significant variation in diagnostic methodology, terminology, and reporting regularity was noted. Despite this limitation, qualitative analysis indicated a continued reduction in the overall number of comorbidities over time and a gradual increase in those with no reported comorbidities. At baseline, there were 37 comorbidities in 15/29 BMI<35 patients. At 3-year follow-up, comorbidities were significantly reduced to 7 (*P* = 0.031); median number of comorbidities per patient fell significantly from 1.0 (0.0-2.0) at baseline to 0.0 (0.0-0.0), *P* = 0.002.

### Quality of life

Three-year postoperative QoL outcomes were available in 58.6% of patients (17/29). Median EQ-5D utility score was 0.8 (0.7-1.0) compared to 0.7 (0.3-0.8) at baseline (Table 3**)**. This represented a significant within-patient median QoL improvement of 0.2 (0.0-0.3) (*P* = 0.028), greater than 2.5 times the accepted clinically important difference. Median EQ-VAS was 75.0 (55.0-90.0) compared to 50.0 (40.0-73.8) at baseline; the median increase of 5.0 (-15.0-40.0) was not significant (*P* = 0.214). Regression analysis indicated a significant association between weight loss and QoL improvement. Using EQ-5D individual change scores as the response variable while controlling for baseline BMI, BMI reduction was significantly related to increasing EQ-5D utility scores (adjusted R^2^ = 0.30; F(2,13) = 4.3; *P* = 0.037).

**Table 3:** Quality of life

QoL Variable	Median (IQR)			*P*-value^†^
	Baseline	3-y	Median change	
EQ-5D	0.7 (0.3-0.8)	0.8 (0.7-1.0)	0.2 (0.0-0.3)	0.028
EQ-VAS	50.0 (40.0-73.8)	75.0 (55.0-90.0)	5.0 (-15.0-40.0)	0.214

*Abbreviations: QoL – Quality of life; EQ-5D – EuroQoL 5-Dimensions; EQ-VAS – EuroQoL-Visual Analogue Scale; IQR – interquartile range.

^†^*P*-values obtained from related-samples Wilcoxon Signed Rank Tests assessing median QoL differences in patients with complete preoperative and 3-y follow-up data (ie, N = 16 for EQ-5D, N = 17 for EQ-VAS).

## Discussion

Results suggest that the SAGB was safe and effective in French patients with a baseline BMI<35. There was no mortality and the AE rate was 0.23 AEs per patient-year, approximately similar to the 0.19 AE rate found in the main HAS cohort study. Adverse events were primarily confined to PBP+SAGB patients; whereas, 83.3% of index SAGB patients experienced no AE. SAGB device survival rate was also comparable to that found in the main cohort study (86.2% vs 87.0%). QoL was improved and a reduction in overall number of comorbidities was observed. BMI reduction was significantly related to positive changes in patient health status. On balance, weight loss trended toward significance at 3 years; however, some patients demonstrated weight gain. For example, those presenting with a BMI<30 (89.0% PBP+SAGB) experienced a median 9.0-kg AW gain (TBWL = -11.8%). Conversely, patients with BMI≥30 to <35 experienced significant AW loss (10.5 kg), median 33.1% EWL, and median 11.0% TBWL – more than double the 5.0% TBWL threshold associated with significant comorbidity improvement (32). First-intervention BMI≥30 to <35 patients experienced a median EWL of 51.0% (TBWL = 17.4%). In addition, within the BMI<35 cohort, logistic regression modeling suggested that a baseline BMI≥30 was the point above which weight loss was likely to occur 3 years post SAGB surgery.

Although weight-loss findings for the BMI≥30 to <35 first-time SAGB patients derive from a very small subgroup (N = 10), their median weight-loss outcomes over 3 years were comparable to those of the 517 morbidly obese patients of the original HAS cohort (median BMI change, 6.1 vs 7.9; EWL, 51.0% vs 49.3%). The subgroup outcomes suggest that surgical weight loss in patients in the BMI≥30 to <35 category follows a pattern similar to that in patients with BMI>35. The observation lends support to the idea that lowering the 1991 NIH (1) bariatric surgery cutoff to 30 may be reasonable. In addition, obesity-related health risks, such as type 2 diabetes mellitus and cardiovascular disease, tend to arise at lower BMIs in certain non-Caucasian populations (eg, Asian Indians) due to a higher percentage and central distribution of body fat (33-35). The Asian Indian Consensus Group, for example, has moved to evaluate weight-related health risk with alternatives to the BMI metric in these patients and to lower the BMI cutoff for bariatric surgery to BMI>32.5 with a comorbidity or BMI>37.5 without comorbidities (36).

The American Society for Metabolic and Bariatric Surgery (ASMBS) Position Statement on BMI 30-35 concluded in late 2012 that class 1 obesity leads to other serious comorbid illnesses and a lowered life expectancy, and that there was no evidence of clinical or cost-effectiveness, ethics, or equity that should exclude the BMI 30-35 group from bariatric surgical treatment (37). The Statement recommended that, at a minimum, certain procedures (ie, gastric banding, sleeve gastrectomy, Roux-en-Y gastric bypass [RYGB]) that have been shown safe and effective in short and mid-term randomized controlled trials in BMI 30-35 patients should be an option for carefully selected patients. O’Brien et al (2006), for example, published a randomized controlled trial of AGB vs medical therapy in BMI 30-35 patients (2 groups of 40 patients each) that demonstrated equivalent weight loss at 6 months; at 2 years, the medical therapy group had regained most of their weight, whereas, the surgical group had an 87.2% EWL (-20 kg) (38). Also, the recently reported randomized controlled “Surgical Therapy and Medications Potentially Eradicate Diabetes Efficiently” (STAMPEDE) trial (2012) showed the effectiveness of sleeve gastrectomy and RYGB in BMI≥27 patients in reducing weight and treating type 2 diabetes mellitus (39).

Evidence for lowering the BMI cutoff for surgery comes from multiple observational studies as well, particularly with respect to the AGB procedure. Angrisani et al (2004) reported the Italian experience in 210 AGB patients with a mean preoperative BMI of 33.9. At 60-month follow-up, mean BMI was 29.2 (40). Parikh et al (2006) described a 26-kg weight loss at 2 years in low-BMI AGB patients that was sustained at 3-year follow-up (41). In 2009, Sultan et al reported 53 AGB patients with a mean baseline BMI of 33.1 who attained a BMI of 25.8 and EWL of 69.7% at 2 years along with substantial improvement in comorbidities (42). Both Choi et al (2010) and Varela et al (2011) compared low-BMI and morbidly obese cohorts undergoing AGB and found the procedure comparably safe and effective in both weight categories (43,44); Varela et al also noted that low-BMI patients had shorter operative times and less blood loss.

In the current SAGB study, in which median weight loss in the BMI≥30 to <35 subgroup was significantly greater than in the BMI<30 subgroup, neither group lost an excessive amount of weight; in fact, mean AW increased slightly in the BMI<30 group (mostly prior bariatric procedure patients), as is typical for bariatric surgery patients after the point of their greatest weight loss. In 2007, Scopinaro et al found in their study of low-BMI biliopancreatic diversion (BPD) patients that, although the mildly obese group lost nearly twice the weight of the overweight group, weight loss was not excessive in either low-BMI category (45). Other surgical studies, including those using BPD, BPD with duodenal switch, AGB, sleeve gastrectomy, and RYGB, have observed the same phenomenon (46-48). Weight loss appears to stabilize within the postoperative year at a BMI>25 regardless of whether the procedure falls into the restrictive, malabsorptive/restrictive, or primarily malabsorptive surgical category (49), and regardless of the preoperative BMI. A homeostatic mechanism may exist that facilitates weight loss in proportion to procedure-specific caloric absorption capacity (5). An integrative analysis of the 16 then-existing bariatric surgery studies in low-BMI patients detected the same pattern of lesser weight loss in patients with BMI<30 than in those with a BMI≥30, suggesting a blunting of the weight-loss cascade at around 30 BMI.

Although the current study was limited by a restricted population of 29, the findings represent a small addition to the evidence base for bariatric surgery in the BMI<35 patients. As in results for the primary HAS “real-world” cohort study, the current low-BMI report contains an underreporting bias partially due to data recording by numerous surgical teams across diverse locations in France; calculating a quantitative measure of change in specific comorbidities was, therefore, not possible.

As early as 1997, Mason et al noted the dramatic trend toward increasingly higher weights in bariatric surgery candidates. They hypothesized that escalating obesity and life-threatening comorbidities should be prevented rather than treated in their full expression (50). Current study outcomes and those of a growing evidence base appear to support the value of lowering the BMI access point for bariatric surgery to permit earlier intervention in appropriate patients. Similar to findings in morbidly obese SAGB patients at 3 years, SAGB treatment for low-BMI patients in France, particularly those with BMI≥30 to <35, was found safe and effective.
